# Two-stage study designs for analyzing disease-associated covariates: linkage thresholds and case-selection strategies

**DOI:** 10.1186/1753-6561-1-s1-s138

**Published:** 2007-12-18

**Authors:** Mike Schmidt, Xuejun Qin, Eden R Martin, Elizabeth R Hauser, Silke Schmidt

**Affiliations:** 1 Center for Human Genetics, Duke University Medical Center, Durham, North Carolina 27710, USA

## Abstract

The incorporation of disease-associated covariates into studies aiming to identify susceptibility genes for complex human traits is a challenging problem. Accounting for such covariates in genetic linkage and association analyses may help reduce the genetic heterogeneity inherent in these complex phenotypes. For Genetic Analysis Workshop 15 (GAW15) Problem 3 simulated data, our goal was to compare the power of several two-stage study designs to identify rheumatoid arthritis-related genes on chromosome 9 (disease severity), 11 (IgM), and 18 (anti-cyclic citrinullated protein), with knowledge of the answers. Five study designs incorporating an initial linkage step, followed by a case-selection scheme and case-control association analysis by logistic regression, were considered. The linkage step was either qualitative-trait linkage analysis as implemented in MERLIN-nonparametric linkage (NPL), or quantitative-trait locus analysis as implemented in MERLIN-REGRESS. A set of cases representing either one case from each available family, one case per linked family (NPL ≥ 0), or one case from each family identified by ordered-subset analysis was chosen for comparison with the full set of 2000 simulated controls. As expected, the performance of these study designs depended on the disease model used to generate the data, especially the simulated allele frequency difference between cases and controls. The quantitative trait loci analysis performed well in identifying these loci, and the power to identify disease-associated alleles was increased by using ordered-subset analysis as a case selection tool.

## Background

There are many possible mechanisms by which environmental or clinical covariates may either influence the risk of complex human diseases directly, or partially account for genetic heterogeneity. For example, they may act as independent environmental risk factors, or increase the disease risk in concert with genetic susceptibility via gene × environment interaction, or define a more homogeneous subgroup of patients in which the main effect of a particular susceptibility gene is more apparent. The purpose of our analysis of the Genetic Analysis Workshop 15 (GAW15) simulated data was to evaluate the power of several two-stage study designs consisting of separate linkage and association analysis steps. These study designs incorporated disease-associated continuous covariates in several different ways. Power comparisons were focused on two distinct factors: 1) thresholds used for the linkage analysis step, which determined the subset of markers included in a subsequent case-control association analysis, and 2) criteria for selecting cases (one per family) to include in this association analysis.

## Methods

With knowledge of the answers, we analyzed the simulated GAW15 microsatellite and SNP data on chromosomes 9, 11, and 18 in an attempt to detect the loci responsible for three disease-associated covariates: disease severity, IgM, and anti-cyclic citrinullated protein (CCP) values, respectively. To investigate Type I error, we also analyzed the relationship of anti-CCP values and genotypes on chromosome 15, which does not harbor any disease-associated loci ("null chromosome"). We analyzed covariate and genotype data from all 1500 nuclear families, and genotype data from all 2000 unrelated controls. We used the MERLIN package [1] to calculate nonparametric multipoint LOD scores for the binary rheumatoid arthritis (RA) affection status [2]. We analyzed the relationship between the family-specific nonparametric linkage (NPL) scores and family averages of the covariates of interest (severity for chromosome 9, IgM for chromosome 11, anti-CCP for chromosome 18) with the ordered-subset analysis (OSA), using the original OSA software [3] and the high-to-low covariate ordering. We also analyzed the covariates themselves as traits in a regression-based quantitative trait locus (QTL) analysis, implemented in MERLIN-REGRESS [4]. IgM values were log-transformed for analysis, and all three covariates were standardized by the sample mean and standard deviation in all genotyped individuals.

We examined five distinct study designs (Table 1), each of which was implemented under two conditions: stringent (LOD score threshold 1.0, OSA *p*-value threshold 0.05, 10-cM region centered on the linkage peak) and loose (LOD score threshold 0.5, OSA *p*-value threshold 0.5, 40-cM linkage region). Each design consisted of two stages. If the first-stage linkage analysis of 1500 families using the microsatellite marker map met the linkage threshold, it was followed by a second-stage association analysis of the SNPs in the linkage region in unrelated cases (one per family) and 2000 controls, using logistic regression with an additive allele coding. The case selection strategies are summarized in Table 1, with Design B being equivalent to the previously proposed "linked best" strategy [5]. The power of each study design to reject the null hypothesis of "no association" (α = 0.05), with or without evidence for linkage, was estimated as the proportion of replicates for which the SNP in highest linkage disequilibrium (LD) with the true disease locus was contained within the linkage region and the case-control association *p*-value from the logistic regression survived the Bonferroni correction for the number of analyzed markers.

**Table 1:** Definition of study designs^a^

Design	Linkage software	Case selection strategy	Notes
A	MERLIN	All	
B	MERLIN	"LINKED BEST" [5]	
C	MERLIN plus OSA	OSA subset if ≥50 families	OSA for localization
D	MERLIN-REGRESS	All	
E	MERLIN-REGRESS (M-R) plus OSA	OSA subset if ≥50 families and difference in OSA and M-R peak ≤20 cM	M-R for localization

^a^Linkage region is centered on maximum LOD score (for all families or OSA subset) and defines SNPs for Stage 2 association analysis.

## Results

Table 2 summarizes characteristics of the loci of interest to illustrate the expected power of linkage and case-control association analyses, respectively. Table 3 shows power estimates for Study Designs A-E for the stringent vs. loose linkage thresholds. A QTL analysis with MERLIN-REGRESS followed by an association analysis of all 1500 cases vs. 2000 controls (Design D) yielded the best results for chromosome 11 (98% power) and 18 (73% power). The chromosome 9 locus was difficult to detect regardless of study design. For chromosome 18, the ability of the OSA-based Designs C and E to detect SNP 269 was greatly improved by using loose linkage thresholds. Most of this effect was due to the thresholds themselves rather than the increased linkage region (data not shown). Of great practical importance, these designs used a much smaller average number of cases in the logistic regression analysis than the most powerful Design D.

**Table 2:** Characteristics of simulated loci

			Average^a^			
				
Chr	Locus (closest SNP)	Distance between true locus and closest SNP (cM)	NPL (SD) at locus^b^	SNP MAF^c ^in cases (one per family, *n *= 1500)	SNP MAF in controls (*n *= 2000)	*r*^2 ^between SNP and true locus
9	G (186)	0.08	0.01 (0.30)	0.374	0.384	0.01
11	F (389)	0.01	0.34 (0.49)	0.275	0.501	0.94
18	E (269)	0.05	0.92 (0.81)	0.297	0.223	0.15

^a^Averages calculated across 100 replicates

^b^NPL, nonparametric multipoint LOD score from MERLIN when analyzing binary RA affection status

^c^MAF, minor allele frequency

**Table 3:** Power estimates for study designs

		Proportion of reps. meeting:							
						
		Linkage threshold		Association threshold (avg. no. SNPs analyzed)^c^		Avg. no. cases analyzed		Overall power	
					
		Stringent^a^	Loose^b^	Stringent	Loose	Stringent	Loose	Stringent	Loose
9 (186)	A	0	0.14	0 (n/a)	0 (144)	1500	1500	0	0
	B	0	0.14	0 (n/a)	0 (144)	983	987	0	0
	C	0.13	0.64	0.23 (48)	0.25 (145)	127	136	0.03	0.16
	D	0.83	0.96	0.01 (48)	0 (147)	1500	1500	0.01	0
	E	0.2	0.65	0.30 (48)	0.20 (148)	129	121	0.06	0.13
									
11 (389)	A	0.06	0.24	1 (52)	1 (147)	1500	1500	0.06	0.24
	B	0.06	0.24	1 (52)	1 (147)	988	985	0.06	0.24
	C	0.11	0.62	1 (48)	0.92 (147)	258	273	0.11	0.57
	D	0.98	1	1 (44)	1 (146)	1500	1500	0.98	1
	E	0.23	0.56	1 (46)	1 (147)	281	290	0.23	0.56
									
18 (269)	A	0.21	0.64	1 (28)	1 (97)	1500	1500	0.21	0.64
	B	0.21	0.64	1 (28)	1 (97)	998	988	0.21	0.64
	C	0.53	0.91	1 (28)	1 (96)	372	390	0.53	0.91
	D	0.73	0.99	1 (27)	1 (95)	1500	1500	0.73	0.99
	E	0.44	0.88	1 (27)	1 (95)	370	382	0.44	0.88

^a^Stringent linkage thresholds are LOD score ≥ 1.0, OSA *p*-value ≤ 0.05, 10-cM linkage region.

^b^Loose thresholds are LOD score ≥ 0.5, OSA *p*-value ≤ 0.5, 40-cM linkage region.

^c^The proportion of replicates meeting the Bonferroni-corrected association threshold, average number of cases and number of SNPs analyzed were calculated across replicates meeting the linkage threshold.

It was previously shown that linkage and association test statistics are statistically independent under the null hypothesis of i) no linkage and no association; ii) linkage and no association; iii) association and no linkage [6]. Consistent with this finding, our analysis of the "null chromosome" (chromosome 15) and the anti-CCP covariate yielded a range of estimated type I error rates from 0 to 0.02 for the stringent thresholds and from 0.01 to 0.05 for the loose thresholds across the five study designs. The range is due to random variation across the limited number of 100 replicates. Because all null hypotheses for the different Stage 1 analyzes are based on linkage statistics, neither LOD score nor OSA *p*-value thresholds affect the type I error rate of the Stage 2 association analysis.

## Discussion

Our study demonstrates that the incorporation of disease-related covariates into a combined linkage and association analysis can help identify genes that contribute directly or indirectly to the risk of RA. Specifically, results for chromosome 18 show that the efficiency of a case-control association analysis can be greatly increased when linkage and covariate information are used to select the cases. For the simulation models used to generate the GAW15 data, the OSA method worked particularly well in this regard because it uses both the family-specific identify-by-descent (IBD) sharing information, and the relationship between covariate distribution and IBD sharing across families to enrich the case sample for the disease allele of interest. For the data sets simulated here, the "linked best" strategy (Design B in our study) was able to achieve the exact same power as Design A with a 34% reduction in the number of analyzed cases, even though it ignored covariate information (Table 3). However, this result does not hold in general [7], and Design A is expected to be most powerful under linkage homogeneity.

The results for SNP 389 on chromosome 11 are not very representative of real data studies. Due to the large minor allele frequency (MAF) difference between cases and controls and almost complete LD with the causal allele (Table 1), a single-stage logistic regression analysis of all SNPs on this chromosome detected the disease-associated SNP in all replicates, even with the conservative Bonferroni correction. This was also the case for SNP 269 on chromosome 18, although it was still possible to compare the efficiency of different designs.

The chromosome 9 data presented two challenges: very weak linkage with respect to affection status, and very small MAF differences between controls and cases pooled across severity categories. The combination of using OSA for case selection, employing loose linkage thresholds, and using MERLIN-REGRESS for localization (Design E) resulted in improved power for the association analysis (from near 0 to 20–30%). However, because OSA used family-specific NPL scores for the binary affection status as input, regardless of disease severity, the overall power of Design E remained low (16% at best). A family-based association analysis of disease severity with the QTDT (quantitative transmission-disequilibrium test) package [8] or a logistic regression analysis comparing only the most severely affected cases (MAF 0.32) with unrelated controls (MAF 0.38) were more powerful analysis approaches for detecting disease severity loci, as simulated here.

## Conclusion

The GAW15 data provided very weak linkage signals for the three loci considered here, presumably due to substantial within-family heterogeneity with respect to the simulated disease loci (Table 2). This made it difficult for a two-stage design to be statistically powerful because stringent linkage thresholds eliminated the association analysis altogether. In this situation, a simultaneous linkage and association analysis with the program LAMP [9] was more successful [10]. Relaxing the linkage thresholds, especially the OSA *p*-value threshold, improved power for chromosome 18, and to a lesser extent chromosome 11, since it identified a subgroup of cases with reduced allelic heterogeneity, even though the linkage evidence in this subgroup continued to be low. In real data sets, a SNP map of the density simulated here is unlikely to include SNPs in high enough LD with susceptibility or quantitative trait loci to detect strong association signals, and the two-stage approach presented here continues to be of practical importance.

## Competing interests

The author(s) declare that they have no competing interests.
